# CUSTODIAL GRANDPARENTS' DEPRESSION AND ACCESS TO HEALTH CARE DURING COVID-19: THE ROLE OF RACIAL DISCRIMINATION

**DOI:** 10.1093/geroni/igac059.1032

**Published:** 2022-12-20

**Authors:** Yanfeng Xu, Theresa Harrison

**Affiliations:** University of South Carolina, Columbia, South Carolina, United States; University of South carolina, Columbia, South Carolina, United States

## Abstract

The COVID-19 pandemic has exacerbated racism against racial minorities and widened racial/ethnic disparities in health outcomes and access to health care services. This study analyzed cross-sectional data (N=219) collected from custodial grandparents via Qualtrics Panels in February 2022 to understand the role of race and perceived racial discrimination in contributing to custodial grandparents’ depressive symptoms and access to health care services. Results indicated that a higher level of perceived racial discrimination was positively associated with grandparents’ more depressive symptoms, but it was also associated with lower odds of custodial grandparents’ access to health care services. Furthermore, racial/ethnic disparities in depressive symptoms and access to telemental health services among custodial grandparents were identified. Results imply the importance of addressing racial/ethnic disparities in depressive symptoms and access to health care services among custodial grandparents.

